# Digestion of the glycosaminoglycan extracellular matrix by chondroitinase ABC supports retinal ganglion cell dendritic preservation in a rodent model of experimental glaucoma

**DOI:** 10.1186/s13041-018-0412-5

**Published:** 2018-11-21

**Authors:** James R. Tribble, Pete A. Williams, Bruce Caterson, Frank Sengpiel, James E. Morgan

**Affiliations:** 1 School of Optometry and Vision Sciences, Cardiff University, Cardiff, Wales CF24 4HQ UK; 2 Department of Clinical Neuroscience, Section of Ophthalmology and Vision, St. Erik Eye Hospital, Karolinska Institutet, Polhemsgatan 50, 112 82 Stockholm, Sweden; 3 School of Biosciences, Cardiff University, Cardiff, Wales CF10 3AX UK; 4 School of Medicine, Cardiff University, Cardiff, Wales CF14 4XW UK

**Keywords:** Retinal ganglion cell, Dendrite, Glaucoma, Neuro-protection, Chondroitinase ABC, Glycosaminoglycan

## Abstract

Retinal ganglion cell dendritic atrophy is an early feature of glaucoma, and the recovery of retinal ganglion cell dendrites is a viable option for vision improvement in glaucoma. Retinal ganglion cell neurites are surrounded by a specialised glycosaminoglycan extracellular matrix which inhibits dendritic plasticity. Since digestion of the extracellular matrix by chondroitinase ABC has been reported to have neuro-regenerative and neuro-plastic effects within the central nervous system, we explored its potential for dendritic recovery in a rat model of ocular hypertension. Chondroitinase ABC was administrated intravitreally 1 week after ocular hypertension (a time point where dendritic atrophy has already occurred). Retinal ganglion cell dendritic morphology was unaffected by chondroitinase ABC in normal retina. In ocular hypertensive eyes retinal ganglion cells showed significantly decreased dendritic length and area under the Sholl curve with atrophy confined to higher order dendrites. These changes were not observed in chondroitinase ABC injected eyes despite similar total retinal ganglion cell loss (i.e. dendritic protection of surviving retinal ganglion cells). These data suggest that glycosaminoglycan digestion could have a therapeutic role in mitigating the effects of elevated pressure on retinal ganglion cell dendritic structure in glaucoma.

**Electronic supplementary material:**

The online version of this article (10.1186/s13041-018-0412-5) contains supplementary material, which is available to authorized users.

## Main text

Glaucoma is a common, age-related, neurodegeneration characterised by the progressive atrophy and death of retinal ganglion cells (the output neuron of the retina). Dendritic atrophy is an early feature of neuronal damage, which has been demonstrated in a number of experimental animal glaucoma models [1–3] and offers an attractive therapeutic window in which neuro-regenerative treatments could restore dendritic connectivity and rescue vision. However, the adult central nervous system has limited plasticity, particularly following injury [4]. The extracellular matrix surrounding neurons in the central nervous system plays a dynamic role in synaptic refinement in the critical period, guiding neurite outgrowth before stabilising favourable connections at its closure [5]. It comprises a hyaluronan backbone, proteoglycan lectican family side chains with glycosaminoglycan (GAG) attachments of varying length and composition [6]. Chondroitin sulphate is a common GAG chain that has demonstrated inhibition of plasticity [7]. When removed through enzymatic digestion with chondroitinase ABC, synaptic plasticity has been enhanced in a number of conditions including shifting ocular dominance [8] and striatal re-innervation following nigrostriatal axotomy [9]. We therefore hypothesized that chondroitinase ABC treatment following glaucomatous degeneration could enable sufficient retinal plasticity for a re-growth of retinal ganglion cell dendrites.

To assess retinal ganglion cell dendritic architecture, we used a rat inducible model of experimental glaucoma in which para-magnetic microbeads were injected into the anterior chamber, and distributed circumferentially with an externally applied magnet to block aqueous humour drainage and elevate intraocular pressure (IOP). Pressure increases typically last 2–3 weeks by which point significant retinal ganglion cell dendritic degeneration has occurred [10, 11]. The left anterior chambers of 19 Brown Norway rats (5 months old) were injected with 30 mg/ml (4.7 × 10^5^ particles) of para-magnetic polystyrene microspheres (4.5 μm diameter, Kisker Biotechnology, Germany) as described by Samsel et al. [10] generating ocular hypertensive eyes (OHT). The IOP was measured before injection and every 3 days subsequently using a rebound tonometer (Tonolab, Icare, Finland). After the return of the IOP to baseline for the period of 1 week, rats received an intravitreal injection in the OHT eye of either: 3 μl chondroitinase ABC (10 U/ml in PBS, from *Proteus vulgaris*, AMS Biotechnology, UK; *n* = 9, OHT ABC group) or vehicle only (1 M PBS, *n* = 10, OHT PBS group). A further 7 rats that were not induced with experimental glaucoma received an intravitreal injection of chondroitinase ABC (10 U/ml in PBS, NT ABC group). All contralateral eyes provided a normotensive un-operated control (NT UOC, *n* = 26). The effect of treatment was assessed 2 weeks after intravitreal injection, allowing sufficient time for dendritic recovery. We confirmed chondroitinase ABC action through immunofluorescent labelling of digested chondroitin sulphate GAGs (2B6 antibody [6]) in the ganglion cell layer (GCL; Additional file 1: Figure S1). Eyes were enucleated and retinas flat mounted. Retinal ganglion cells were labelled DiOlistically as described previously [11]. Z-stack confocal images were acquired and dendritic arbours traced manually using NeuronJ [12] in FIJI [13]. TOPRO-3 cell counts were performed on 4 regions of the GCL (100 μm^2^ *en face* area, 1500 μm from the optic disc nasally, temporally, superiorly and inferiorly) and averaged. Statistical analysis was performed in R; all tests are one-way ANOVA with Tukey HSD.

**Fig. 1:**
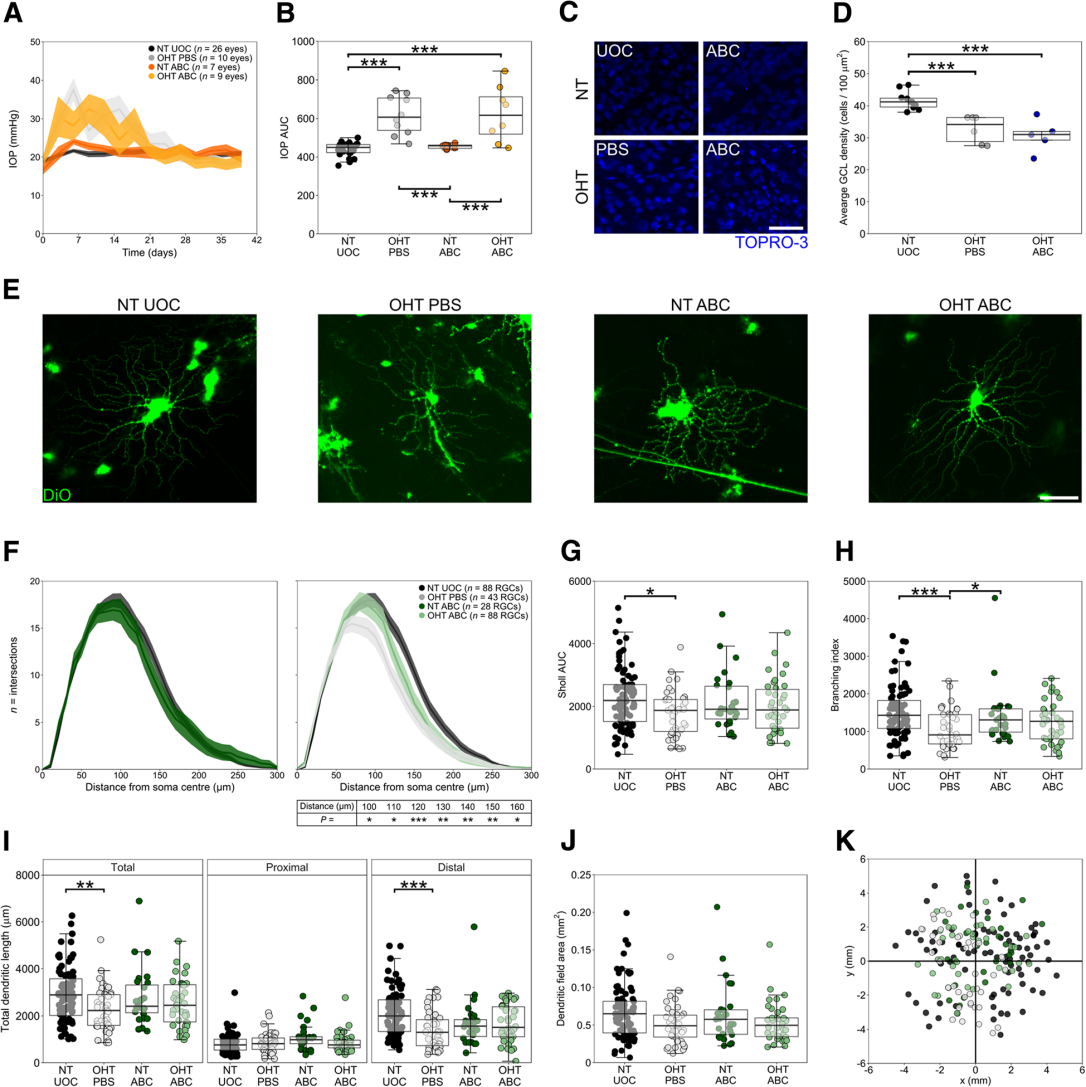
Chondroitinase ABC protects dendritic architecture following ocular hypertension. **a** Microbead injections produced 2–3 weeks of raised IOP; average profiles with shaded SD shown. **b** The AUC of IOP profiles shows a significantly greater IOP in OHT groups compared to NT UOC. Chondroitinase ABC had no effect on IOP post injection. **c** Cell loss was assessed by TOPRO-3 counts; example images shown. **d** OHT resulted in significant cell loss in the GCL compared to NT UOC; cell loss was not significantly different between OHT groups. **e** Dendritic morphology was assessed following DiOlistic labelling; example cells from each group shown. **f** Sholl profiles demonstrate that chondroitinase ABC injection in NT ABC eyes had no effect on dendritic complexity (*left*). OHT PBS eyes showed a reduced complexity (statistical values shown in table below plot for NT UOC against OHT PBS) that was rescued by chondroitinase ABC injection (*right*). Analysis of dendritic morphology revealed a significant reduction in OHT PBS retinal ganglion cells compared to in NT UOC for metrics of Sholl AUC (**g**), branching index (**h**), total dendritic length and distal dendritic length (**i**). **j** Dendritic field area was unchanged across groups, indicating reduced density of branches. **k** Retinal ganglion cell eccentricity had no effect on dendrite morphology, with cells analyzed across all regions (optic nerve head at *x =* 0, *y* = 0; group colors as in F-J). Multivariate regression analysis showed no significant correlation between the distance from optic disc and the outcome variables (Sholl AUC: R^2^ 0.0018, *P* = 0.25; Sholl BI: R^2^–0.0026, *P* = 0.47; dendritic field area: R^2^ 0.0993, *P* = 0.01, total dendritic length: R^2^ 0.0033, *P* = 0.56, proximal dendritic length: R^2^ 0.0087, *P* = 0.10, and distal dendritic length: R^2^ 0.0195, *P* = 0.03). * = *P* < 0.05, ** = *P* < 0.01, *** = *P* < 0.001. Scale bars = 50 μm for (**c**), 100 μm for (**e**). For box plots, center hinge represents the mean and the upper and lower hinges represent the first and third quartiles; whiskers represent 1.5 times the interquartile range

OHT eyes demonstrated increased IOPs for 2–3 weeks (mean ± SD = 20 ± 5 days for OHT PBS, 18 ± 4 days for OHT ABC; Fig. 1a) which was significantly greater (IOP area under curve (AUC)) than contralateral controls (NT UOC; *P* < 0.001 for OHT PBS, < 0.001 for OHT ABC; Fig. 1b). Chondroitinase ABC injection alone had no effect on IOP (AUC, *P* = 0.972). Cell counts revealed a significant decrease in cells in the GCL in both OHT PBS (21% loss, *P* < 0.001) and OHT ABC-treated eyes (26% loss, *P* < 0.001) when compared to contralateral control eyes (Fig. 1c-d). The degree of cell loss was not significantly different between OHT PBS and OHT ABC (*P* = 0.197) indicating comparable IOP induced damage. Analysis of dendritic trees revealed that chondroitinase ABC conferred a significant protection of dendrites in glaucoma (Fig. 1e for representative cells). Sholl analysis (a measure of dendrite frequency at binned distance from the soma, Fig. 1f) revealed a reduced dendritic complexity in OHT PBS as demonstrated by significantly fewer dendrites at distances of 100-160 μm from the soma centre (*P* < 0.05, see Fig. 1f for individual values). These Sholl changes were also confirmed when expressed as an AUC (*P* = 0.014; Fig. 1g) and as a branching index (branching events weighted by distance from soma, [14]; *P* < 0.001; Fig. 1h). This dendritic atrophy was not observed in OHT ABC (AUC *P* = 0.578, branching index *P* = 0.086). Total dendritic length was decreased in OHT PBS (*P* = 0.006) but not OHT ABC (*P* = 0.365) compared to NT UOC (Fig. 1i). Analysis of individual dendrites revealed that this was confined to the distal dendrites (quaternary and above; *P* < 0.001) with no changes in proximal dendrites (primary, secondary, tertiary; *P* = 0.901). Dendritic field area was unchanged between groups; dendrite loss in the absence of filed size changes is indicative of a loss of dendritic density (Fig. 1j). Retinal ganglion cells were sampled evenly across the retina (Fig. 1k) and thus the eccentricity did not impact morphological comparisons.

These data demonstrate moderate dendritic preservation in ocular hypertensive eyes following chondroitinase ABC treatment. Further studies in which single cells are followed longitudinally over time are required to confirm whether this is a protective or regenerative effect. Since it is well established that dendritic loss occurs early during ocular hypertensive insult [3], and as we have previously reported significant dendritic loss in this model after 2 weeks of raised IOP [11] the delayed nature of the treatment in this experimental paradigm is suggestive of a regrowth of dendrites, however, additional time points are required to fully assess this.. Removal of chondroitin sulphate GAG chains may therefore have increased the plastic potential of the retina following injury and may be of therapeutic benefit in glaucoma. In a recent study, the use of Arylsulfatase B to cleave only inhibitory 4-sulphated chondroitin sulphate chains facilitated increased axon outgrowth following optic nerve crush [15]. Therefore a more targeted removal of chondroitin sulphate chains may prove to be more efficacious in glaucoma.

## Additional file

Additional file 1: **Figure S1.** Chondroitinase ABC action in the GCL. Chondroitinase ABC digestion of chondroitin sulphate GAG sidechains leaves ‘stubs’ that can be targeted by immunofluorescent labelling of cryosections. Digestion to a sulphation residue exposes antibody binding sites for 2-B-6 (targeting 4-sulpahted N-acetylgalactosamine). 2-B-6 labelling (green) is clear surrounding cells in the GCL (white; nuclei stained with Hoechst-33,342) in chondroitinase ABC injected eyes (OHT ABC) and is absent in OHT PBS and negative controls sections (2° antibody only). Choroid incubated with chondroitinase ABC ex vivo is used as a positive control, in which 2-B-6 labelling is clear. Scale bar = 50 μm for retinal sections, 25 μm for choroid section. (TIF 5737 kb)

## Abbreviations

AUC: Area under the curve
GAG: Glycosaminoglycan
GCL: Ganglion cell layer
IOP: Intraocular pressure
NT UOC: Normotensive un-operated control
OHT ABC: Ocular hypertensive chondroitinase ABC treated
OHT PBS: Ocular hypertensive phosphate buffered saline treated
OHT: Ocular hypertensive
PBS: Phosphate buffered saline
