# Supplementary figures and images for: Digestion of the glycosaminoglycan extracellular matrix by chondroitinase ABC supports retinal ganglion cell dendritic preservation in a rodent model of experimental glaucoma
Source: Mol Brain. 2018 Nov 21;11:69. doi: 10.1186/s13041-018-0412-5 (PMC6249825; doi:10.1186/s13041-018-0412-5)

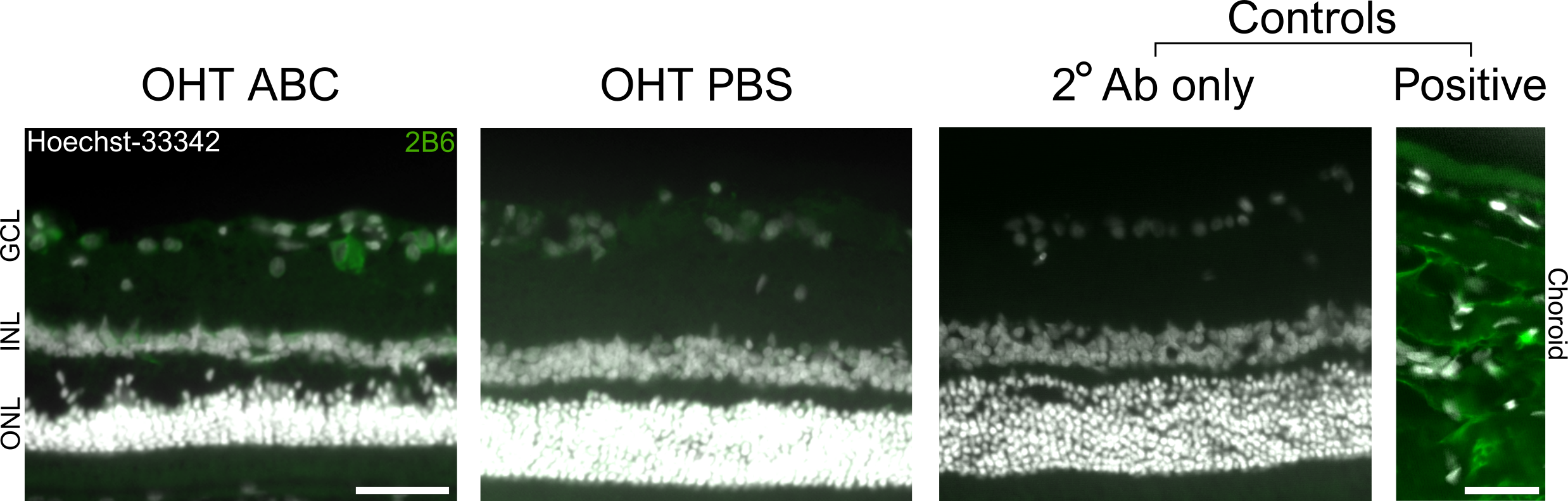

Supplement: Supplementary file 1 — Figure S1. Chondroitinase ABC action in the GCL. Chondroitinase ABC digestion of chondroitin sulphate GAG sidechains leaves ‘stubs’ that can be targeted by immunofluorescent labelling of cryosections. Digestion to a sulphation residue exposes antibody binding sites for 2-B-6 (targeting 4-sulpahted N-acetylgalactosamine). 2-B-6 labelling (green) is clear surrounding cells in the GCL (white; nuclei stained with Hoechst-33,342) in chondroitinase ABC injected eyes (OHT ABC) and is absent in OHT PBS and negative controls sections (2° antibody only). Choroid incubated with chondroitinase ABC ex vivo is used as a positive control, in which 2-B-6 labelling is clear. Scale bar = 50 μm for retinal sections, 25 μm for choroid section. (TIF 5737 kb) [file 13041_2018_412_MOESM1_ESM.tif]
